# Research progress on extraction, purification, structure and biological activity of *Dendrobium officinale* polysaccharides

**DOI:** 10.3389/fnut.2022.965073

**Published:** 2022-07-18

**Authors:** Yuan He, Lin Li, Hao Chang, Bin Cai, Huajun Gao, Guoyu Chen, Wen Hou, Zubaydan Jappar, Yizhe Yan

**Affiliations:** ^1^College of Food and Bioengineering, Henan Key Laboratory of Cold Chain Food Quality and Safety Control, Zhengzhou University of Light Industry, Zhengzhou, China; ^2^College of Life Sciences, Zhengzhou Normal University, Zhengzhou, China; ^3^Cigar Research Institute, Anhui Tobacco Technology Center, Bengbu, China; ^4^Haikou Cigar Research Institute, Hainan Provincial Branch of CNTC, Haikou, China

**Keywords:** *Dendrobium officinale*, polysaccharides, structural characterization, biological activity, structure-activity relationship

## Abstract

*Dendrobium officinale* Kimura et Migo (*D. officinale*) is a traditional medicinal and food homologous plant that has been used for thousands of years in folk medicine and nutritious food. Recent studies have shown that polysaccharide is one of the main biologically active components in *D. officinale*. *D. officinale* polysaccharides possess several biological activities, such as anti-oxidant, heptatoprotective, immunomodulatory, gastrointestinal protection, hypoglycemic, and anti-tumor activities. In the past decade, polysaccharides have been isolated from *D. officinale* by physical and enzymatic methods and have been subjected to structural characterization and activity studies. Progress in extraction, purification, structural characterization, bioactivity, structure-activity relationship, and possible bioactivity mechanism of polysaccharides *D. officinale* were reviewed. In order to provide reference for the in-depth study of *D. officinale* polysaccharides and the application in functional food and biomedical research.

## Introduction

*Dendrobium officinale* Kimura et Migo (*D. officinale*), commonly known as *Tiepi Shihu*, belongs to the orchid family *Dendrobium* genus (1), largely distributed in tropical and subtropical regions, especially in southern China, Japan, India, Australia, and other regions (2). There are more than 1,500 species of *Dendrobium* in the world, and in China has reported more than 80 species of *Dendrobium*.

*Dendrobium* is a precious Chinese herbal medicine with a long history of medicinal use in China. Modern pharmacological studies show that *D. officinale* has anti-inflammatory, nourishing the spleen and stomach, immunomodulatory, and prolonging life effects (3). Importantly, *D. officinale* is rich in a variety of bioactive components, such as polyphenols, flavonoids, alkaloids, polysaccharides, amino acids, and vitamins, etc., of which polysaccharides are considered to be the main active substances in *D. officinale* (4–8). *D. officinale* polysaccharides obtained by different extraction and separation methods have different physicochemical properties and biological activities, including anti-cancer (9), anti-inflammatory (10), antioxidant (11), hypoglycemic (12), immune regulation (13), gastrointestinal tract protection (14), and liver protection (15) effects.

In recent years, some scholars have reviewed *D. officinale* polysaccharides. For example, Chen et al. (1) reviewed the research progress on the separation, structural properties and biological activities of *D. officinale* polysaccharides. Yue et al. (16) summarized the isolation methods, structural properties, and biological activities of *D. officinale* polysaccharides. Li et al. (17) systematically summarized the preparation, structural characterization and bioactive molecular mechanism of *D. officinale* polysaccharides. However, there is a lack of systematic overview of the structural characteristics, biological activity mechanism, and structure-activity relationship of *D. officinale* polysaccharides. Therefore, this paper systematically reviews the extraction, separation, purification, structural properties, biological activities, and mechanism of action of *D. officinale* polysaccharides, and focuses on structure-activity relationship of *D. officinale* polysaccharides.

## Extraction, isolation, and purification methods

**FIGURE 1:**
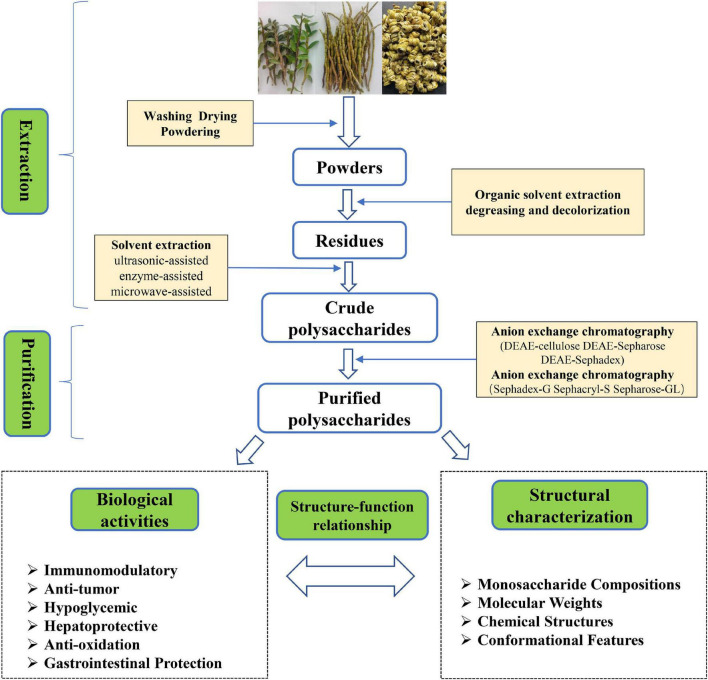
Schematic representation of the extraction, purification, and bioactivity of polysaccharides from *Dendrobium officinale*.

To obtain more bioactive polysaccharide from *D. officinale*, it needs to be extracted, separated and purified as shown in Figure 1. First, fresh *D. officinale* was washed and dried at low temperature and ground into powder. Next, the powder was soaked in a water bath (70°C) for 3 h in 90–95% ethanol to inactivate the enzyme and remove small molecular substances such as oligosaccharides, amino acids, lipids, and pigments (18). Then, the ethanol is filtered and evaporated to dryness. Finally, the solid residue is extracted by different methods, for example hot water extraction (19), ultrasonic-assisted extraction (20), enzyme-assisted extraction (21), microwave-assisted extraction (22), supercritical fluid extraction (23), and freeze-thaw cold pressing (24).

Hot water extraction is the most commonly used extraction method in laboratory and industry because it is simple, convenient and safe, but hot water extraction is long and inefficient. Other methods are also usually used to assist extraction, such as ultrasonic-assisted extraction and microwave-assisted extraction. He et al. (24) conducted experiments for comparing the extraction of polysaccharides from hot water, ultrasonic assisted hot water extraction, and microwave assisted hot water extraction, the yield of polysaccharides from *D. officinale* was the highest at 20.55%, followed by microwave-assisted hot-water extraction at 17.74%. Ultrasound and microwave could damage the cell wall, causing an outflow of substances within the cell, which increases the efficiency of polysaccharide extraction, but long-term use leads to structural destruction of polysaccharides (25). In addition to the physical extraction method, enzymes are also used to assist the extraction of *D. officinale* polysaccharides. Currently, the enzymes used to prepare polysaccharides mainly include cellulase, pectinase, and protease. Enzymes could soften the cell wall, change the permeability of the cell wall, and make the cell contents easier to dissolve, thereby improving the extraction efficiency (26). He et al. (24) used cellulase-assisted extraction of *D. officinale* stem polysaccharides, and the extraction rate was 18.50%, which was much higher than that of hot water extraction. To sum up, the hot water extraction method is simple and convenient, but the extraction rate is low and time-consuming; ultrasonic-assisted, and microwave-assisted extraction methods can improve the extraction efficiency, but long-term ultrasonic or microwave irradiation could degrade polysaccharides and change the structure of polysaccharides; enzyme-assisted extraction method has the advantages of mild reaction conditions and high efficiency, but it has high production costs and strict environmental requirements.

It is worth mentioning that the yield of *D. officinale* polysaccharides is not only related to the extraction method, but also to the extraction conditions (such as solid-liquid ratio, extraction time, and extraction temperature). The different extraction conditions of polysaccharides were optimized by response surface methodology and orthogonal method. For example, Pan et al. (27) found that the optimal enzyme-assisted extraction conditions of *D. officinale* polysaccharides were: solid-liquid ratio 1:25, pH 5.5, cellulase 10 m/L, extraction temperature at 40°C and extraction time of 3 h, the polysaccharide yield was 8.41 g/100 g dry weight, which was 1.25 times that of hot water extraction. Guo et al. (20) found that when the ultrasonic power was 144 W, the extraction time was 150 min, the extraction temperature was 353.15 K, and the solid-liquid ratio was 60:1, the highest yield of *D. officinale* polysaccharide was 43.7%.

The crude extract of *D. officinale* polysaccharide usually contains some impurities such as pigments, proteins, and other small molecules. Therefore, decolorization and deproteinization are the keys to purify *D. officinale* polysaccharide. At present, the decolorization of *D. officinale* polysaccharide is mainly carried out by activated carbon adsorption method, hydrogen peroxide method, and resin adsorption method (1). Among different decolorization methods, the hydrogen peroxide method has the best decolorization effect, but the amount of hydrogen peroxide should be strictly controlled, otherwise it will destroy the polysaccharide structure and even lead to polysaccharide degradation. Compared with the oxidation method, the activated carbon adsorption method has little effect on the polysaccharide structure, but due to its porous structure, the polysaccharide retention rate was low. Macroporous resin adsorption has high stability and selective adsorption, which is an ideal decolorization method for polysaccharides (28–30). Till now, the protein in polysaccharide is mainly removed by Sevag and trichloroacetic acid. Among them, trichloroacetic acid has the best protein removal effect and high polysaccharide retention rate, but excessive trichloroacetic acid will lead to polysaccharide degradation. The Sevag reagent is often used for deproteinization of *D. officinale* polysaccharides due to its mild reaction. However, the Sevag reagent has low deproteinization efficiency, low polysaccharide retention rate and toxic reagent residues (17, 31, 32). In the future, safer and more environmentally friendly methods such as enzymatic hydrolysis, resin adsorption, and ultrafiltration could be used to deproteinize *D. officinale* polysaccharides.

It is reported that *D. officinale* polysaccharide is a heteropolysaccharide containing different components, and the separation of *D. officinale* polysaccharide into a single component is the basis for studying the chemical structure (33). To date, step-by-step alcohol precipitation has become a method for the initial separation of polysaccharides. As the molecular weight of polysaccharides increases, the solubility of polysaccharides in ethanol decreases. Based on this principle, different concentrations of ethanol are used to separate polysaccharides of different components (34). Xing et al. (35) successfully separated four components from DOP by fractional alcohol precipitation (40, 50, 60, and 70% v/v) method. Although the fractional precipitation method is simple, the molecular weight distribution of the polysaccharide after separation is wide, which is a rough separation method. In addition, column chromatography is a commonly used method for the separation and purification of polysaccharides. It could be divided into anion exchange chromatography and gel filtration chromatography. Neutral polysaccharides and acidic polysaccharides were separated by anion exchange chromatography with different concentrations of NaCl, and polysaccharides of different molecular weight were separated by gel filtration chromatography (36, 37). Currently, anion chromatography columns (DEAE-cellulose and DEAE-Sepharose Fast Flow) separated acidic polysaccharides and medium polysaccharides according to polysaccharide polarity (37–39). Gel filtration chromatography (Sephadex-G series and sephacryl-S series) separated polysaccharides based on molecular weight differences (40–42). For instance, Xie et al. (39) used a DEAE-Cellulose column (1.6 cm × 60 cm) to elute with water and 0.05 M NaCl solution to obtain two separated fractions (DOP-W and DOP-S). Sun et al. (43) isolated three major polysaccharide components (DCPP-I, DCPP-I-a, and DCPP-II) from DCPP by DEAE-Cellulose-52 and Sephadex G-200 column, wherein DCPP-I-a was composed of xylose, glucose, galactose was composed in a molar ratio of 1.44:6.93:12.79 and had a molecular weight of 67 KDa. Besides column chromatography, other purification techniques such as salting out, ultrafiltration, metal coordination are also used for polysaccharide purification (37).

## Physiochemical and structural characterization

The physicochemical structural characteristics of plant polysaccharides mainly include monosaccharide composition, molecular weight, chemical structures, type and position of glycosidic bonds, and spatial conformation (44). A variety of polysaccharide components were isolated from *D. officinale* polysaccharides, and their chemical structures could be determined by a combination of chemical analysis, spectroscopic analysis, and chromatographic analysis, such as high performance gel permeation chromatography (HPGPC), high-performance liquid chromatography (HPLC), gas chromatography (GC), gas chromatography-mass spectroscopy (GC-MS), fourier transform infrared spectroscopy (FT-IR), nuclear magnetic resonance (NMR), scanning electron microscope (SEM), atomic force microscope (AFM), methylation analysis, Smith degradation, periodate oxidation, etc (37, 44–46). The structural characteristics of *D. officinale* polysaccharides such as monosaccharide composition, molecular weight, chemical structure and biological activity are summarized in Table 1.

**TABLE 1:** The polysaccharides isolated from *Dendrobium officinale*.

No.	Compound name	Source	Mw (Da)	Monosaccharide composition	Structures	Biological activities	References
1	DOP-W3-b	Stems of *D. officinale*	1.543 × 10^4^	Man, Glc in the ratio of 4.5:1.0	Backbone consisting of β-(1→4)-D-Man*p*, β-(1→4)-D-Glc*p* and β-(1→3,6)-D-Man*p* residues; branch consisting of β-(1→4)-D-Man*p*, β-(1→4)-D-Glc*p* and terminal β-D-Glc*p*, and O-acetyl groups attached to 2-O-2 of β-(1→4)-D-Man*p*	Immunoregulation	39
2	DPOa	*Tiepi Fengdou*	8.1 × 10^5^	Man, Glc in the ratio of 5.6:1.0	Backbone consisting of β-(1→4)-D-Man*p*, β-(1→4)-D-Glc*p* residues	Immunoregulation	47
3	DOPb	*Tiepi Fengdou*	6.7 × 10^5^	Man, Glc in the ratio of 5.9:1.0	Backbone consisting of β-(1→4)-D-Man*p*, β-(1→4)-D-Glc*p* residues	Immunoregulation	
4	DOP	Stems of *D. officinale*	8.5 × 10^3^	Man, Glc, Ara, GalA in the ratio of 6.2:2.3:2.1:0.1	Backbone consisting of β-(1→4)-D-Man*p*, β-(1→4)-D-Glc*p* residues	Antioxidant	41
5	HPS-1B23	Herba *Dendrobii*	2.2 × 10^4^	Glc, Man, Gal in the ratio of 31:10:8	Backbone consisting of α-(1→6)-D-Glu*p*, α-(1→4)-D-Glc*p*,α-(1→3,6)-D-Man*p*	Immunoregulation	42
6	FP	*D. officinale*	2.53 × 10^3^	Glc, Gal, Man in the ratio of 2.1:3.4:3.9	Backbone consisting of →3,6)-β-L-Man*p*-(1→, α-D-Glc*p*-(1→, →4)-α-D-Glc*p*-(1→, →3,6)-β-D-Gal*p*-(1→, →6)-β-D-Gal*p*-(1→	Antioxidant, immunoregulation	48
7	DOPS-1	*D. officinale*	1.57 × 10^6^		Backbone consisting of (1→4)-β-D-Glc*p*, (1→4)-β-D-Man*p* and 2-O-acetyl-(1→4)-β-D-Man*p*	Antioxidant, antitumor	40
8	DDFPs50	*D. devonianum*	5.63 × 10^5^	Man, Glc, Gal, Rha, Ara, Fru, GlcA in the ratio of 8.45:2.93:1.00:0.06:0.37:0.04:0.2		Antioxidant	34
9	DOPA-1		2.29 × 10^5^	Man, Glc, Gal in the ratio of 1:0.42:0.27	contain (1 → 3), (1 → 2), and (1 → 6) linkages in the main or branch chains	Anti-tumor	49
10	DOPA-1	Fresh stems of *D. officinale*	3.94 × 10^5^	Man, Glc in the ratio of 5.8:1	Backbone consisting of β-(1→4)-D-Man*p*, β-(1→4)-D-Glc*p* residues	Antioxidant, immunoregulation	5
11	DOPA-2	Fresh stems of *D. officinale*	3.62 × 10^5^	Man, Glc in the ratio of 4.5:1	Backbone consisting of β-(1→4)-D-Man*p*, β-(1→4)-D-Glc*p* residues	Antioxidant, immunoregulation	
12	DOP-1	*D. officinale*	6.8 × 10^3^	Man, Glc in the ratio of 5.18:1	Backbone consisted of →4)-β-D-Glc*p*-(1→, →4)-β-D-Man*p*-(1→, →4)-2-O-acetyl-β-D-Man*p*-(1→ and →4)-3-O-acetyl-β-D-Man*p*-(1→	Hypoglycemic	50
13	DOP-2	*D. officinale*	1.43 × 10^4^	Man, Glc in the ratio of 4.78:1	Backbone consisted of →4)-β-D-Glc*p*-(1→, →4)-β-D-Man*p*-(1→, →4)-2-O-acetyl-β-D-Man*p*-(1→ and →4)-3-O-acetyl-β-D-Man*p*-(1→	Hypoglycemic	
14	DLP- 1	*D. officinale* leaves	1.38 × 10^6^	Man, Glc in the ratio of 3.13:1	Backbone consisted of (1→4)-β-D- Man*p*, (1→4)-β-D- Glc*p*, and (1→4)-2-O- acetyl-β-D-Man*p*	Immunoregulation	51
15	DLP- 2	*D. officinale* leaves	1.93 × 10^6^	Rha, Ara, Gal in the ratio of 1.37:0.94:1	Backbone consisted of (1→4)-β-D-Man*p*, (1→4)-β-D-Glc*p*	Immunoregulation	
16	DOP1-1	*D. officinale*	1.78 × 10^5^	Man, Gal in the ratio of 5.9:1	*O*-acetylated glucomannan with β-D configuration	Immunoregulation	52
17	DCP	Fresh *D. catenatum* stems	2.21 × 10^5^	Glc, Man, GalA in the ratio of 30.2:69.5:0.3	Backbone consisted of (1→4)-β-D-Man*p*, 2-*O*-acetyl-(1→4)-β-D-Man*p*, (1→6)-α-D-Glc*p*, and (1→4)-α-D-Glc*p* residues	Immunoregulation	53
18	LDOP	Leaf of *D. officinale*	9.18 × 10^4^	Man, Gla, Glc, GlcA, and Ara in the ratio of 2.0:1.3:1.6:1.7:0.7	Backbone consisting of α-(1→4)-D-Man*p*, α-(1→6)-D-Glc*p*	Anti-inflammatory	54
19	DOA1a	Dried stems of *D. officinale*	3.7 × 10^4^	Ara, Xyl, Glc, 4-*O*-methylglucuronic acid(4-MGA), Rha, Gal in the ratio of 8.9: 62.7: 8.5: 12.3: 3.9: 3.7	Backbone consisting of β-(1→4)-D-xylan; branch consisting of α-(1→4)-D-Glc*p*, α-(1→3)-L-Rha*p*	Anti-angiogenesis	55
20	DOP-1	*D. officinale* Kimura et Migo	4.47 × 10^5^	Gal, Glc, Man in the ratio of 1:1.79:6.71	Backbone consisting of →4)-α-D-Glc*p*-(1→4)-α-D-Man*p*-(1→4)-α-D-Man*p*-(1→4,6)-α-D-Man*p*-(1→	Antioxidant, anti-tumor	56

### Monosaccharide compositions

In the research on the structural characteristics, physicochemical properties, and structure-activity relationship of plant polysaccharides, the composition of monosaccharides is the most basic and core research object. In most cases, analysis of monosaccharide composition includes polysaccharide hydrolysis, chemical modification, and quantitative detection by HPLC, GC (44, 57). The monosaccharide composition of polysaccharides obtained from different varieties, origins and extraction methods is obviously different. However, most *D. officinale* polysaccharides consisted mainly of glucose, mannose, and galactose according to different moles (1). In one study, for example, Huang et al. (5) purified two polysaccharides (DOPA-1 and DOPA-2) through Sephacryl S-300 chromatography with different molar ratios of mannose to glucose as shown in Table 1. Meanwhile, Tian et al. (58) compared before and after fermentation, the monosaccharide composition *D. officinale* polysaccharide changed little. Interestingly, He et al. (24) obtained *D. officinale* polysaccharides by different extraction methods, which consisted of different molar ratios of mannose and glucose.

### Molecular weights

Molecular weight of polysaccharide is usually determined by HPLC, HPGPC, and high performance size exclusion chromatography multiangle laser light scattering (HPSEC-MALLS) (29, 33). It could be concluded from Table 1 that the molecular weight distribution of *D. officinale* polysaccharides was wide, and the molecular weight ranged from 2.53 to 1,930 kDa. At the same time, the molecular weight of *D. officinale* polysaccharides was affected by the source of raw materials and the method of extraction, separation, and purification. Zhang et al. (10) used Sephadex G-100 column to separate two polysaccharides (DLP-1, DLP-2) from *D. officinale* leaves with molecular weights of 28.342 and 41.143 kDa. Comparing the effect of different extraction methods on the molecular weight of *D. officinale* polysaccharide, compared with hot water extraction and enzyme-assisted extraction, ultrasonic-assisted extraction had the lowest molecular weight (197.1 kDa), because long time ultrasonic extraction could destroy the polysaccharide chain structure and cause polysaccharide degradation (24).

### Chemical structures

**FIGURE 2:**
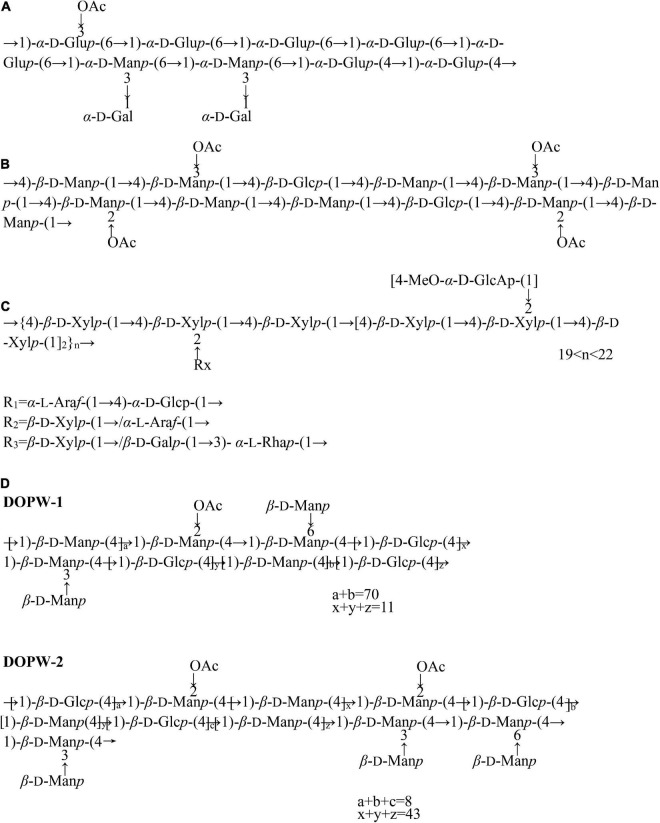
The chemical structure of *D. officinale* polysaccharides. **(A)** The *D. huoshanense* polysaccharide (HPS-1B23), **(B)** two component polysaccharides (DOP-1, DOP-2), **(C)** a novel polysaccharide (S32), **(D)** two novel polysaccharide fractions (DOW-1, DOPW-2).

In addition to the research on the monosaccharide composition and average molecular weight of *D. officinale* polysaccharides, the chemical structure characterization of polysaccharides has also gradually attracted attention (Table 1). At present, the chemical structures of polysaccharides have been analyzed by methylation analysis, periodic acid oxidation, Smith degradation, GC-MS, infrared spectroscopy, FT-IR, and NMR (32, 37, 57). So far, several studies have reported the chemical structure of *D. officinale* polysaccharides (Figure 2). Zha et al. (42) elucidated the structure of a polysaccharide (HPS-1B23) isolated by DEAE-Cellulose anion-exchange column, Sephacryl S-200 column, Sephadex G-75 column. According to the results of periodate oxidation, methylation analysis and NMR, the repeating unit of HPS-1B23 was constructed as shown in Figure 2A. They also found that the C-3 position of the (1→6)-linked glucose was partially substituted by an acetyl group. And the molar ratio of (1→6)-linked glucose, (1→4)-linked glucose, (1→3,6)-linked mannose, and (1→3,6)-linked mannose in backbone is 4:2:1:2.2.

Kuang et al. (50) characterized the structure of two component polysaccharides (DOP-1, DOP-2) isolated from *D. officinale*. Studies have shown that both DOP-1 and DOP-2 were composed of mannose and glucose, and it is speculated that their backbone structures are both composed of →4)-β-D-Glc*p*-(1→, →4)-β-D-Man*p*-(1→, →4)-2-O-acety-β-D-Man*p*-(1→ (Figure 2B).

Yue et al. (55) isolated a novel polysaccharide (S32) from the crude polysaccharide of *D. officinale* extracted with DEAE Sepharose Fast Flow and Sephacryl S-300. Studies have shown that it was composed of arabinose, xylose, glucose, 4-MGA, and a small amount of galactose and rhamnose. We infer that the structural repeat unit of S32 was shown in Figure 2C, and it contained a backbone of 1,4-linked β-D-xylan, with branches of 1,4-linked α-D-glucose, 1,3-linked α-L-rhamnose, and terminal-linked α-L- arabinose, β-D-galactose, 4-MGA, and β-D-xylose directly or indirectly attached to C-2 position of glycosyl residues on backbone (55).

Tao et al. (59) isolated two polysaccharide components (DOW-1 and DOPW-2) from *D. officinale*, and characterized the polysaccharide structure based on methylation analysis and NMR, inferring the repeating unit structure of DOPW-1 and DOPW-2 (Figure 2D). Meanwhile, methylation analysis revealed that both DOPW-1 and DOPW-2 possessed the same backbones of →4)-β-D-Man*p*-(1→, →4)-β-D-Glc*p*-(1→, although with different percentages.

He et al. (52) isolated a fraction of neutral heteropolysaccharide (DOP1-1) from *D. officinale* and analyzed their physicochemical properties. NMR and FT-IR analysis indicated that partial structure of DOP-1-1 is an *O*-acetylated glucomannan with β-D configuration in pyranose sugar forms.

### Conformational features

Till now, the primary structure of *D. officinale* polysaccharide has been studied by lots of experiments, but there were few reports on the solution properties and conformation features of *D. officinale* polysaccharide (1). At present, the advanced structure characterization of polysaccharide mainly includes Congo red experiment, SEM, AFM, circular dichroism, and X-ray diffraction (XRD) (36, 44). Zhong et al. (51) isolated two polysaccharides (DLP-1 and DLP-2) from *D. officinale* leaves, both of which could form complexes with Congo red in the Congo red experiment, indicating that DLP-1 and DLP-2 had triple helices structure. Besides, the surface structure of DLP-1 was irregular large flakes with smooth surface and porous interior. In contrast, the surface of DLP-2 was rough, loose, and uneven, consisting of large spongy particles, which might be due to the binding of polysaccharides to different macromolecules and the freeze-drying and dehydration processes affecting the morphology of polysaccharides. In the future, more advanced technology is needed to conduct research in order to better understand the structure-activity relationship of *D. officinale* polysaccharides.

## Biological activities

**FIGURE 3:**
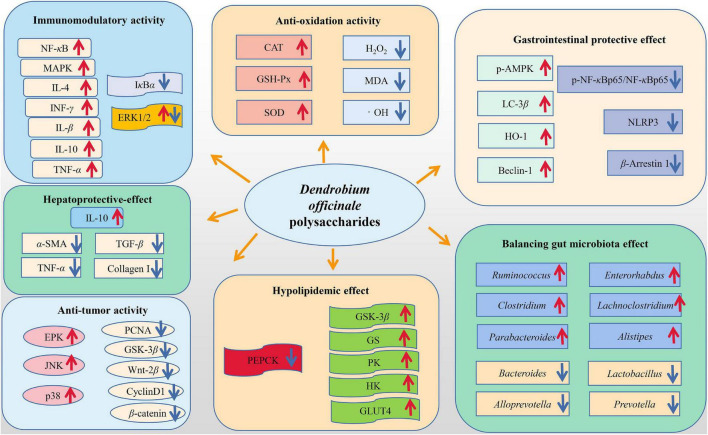
Bioactivities of *D. officinale* polysaccharides.

In recent years, *D. officinale* polysaccharides have attracted extensive attention in the field of biological and medical for their various biological properties and pharmacological functions. Studies *in vitro* and *in vivo* have shown that *D. officinale* polysaccharides have a variety of pharmacological activities, including immunomodulatory (60), antioxidant (56), anti-tumor (49), gastrointestinal protection (61), anti-aging (3), hepatoprotective (62), hypoglycemic (50), and anti-inflammatory (63). Among them, immune regulation, anti-oxidation, anti-cancer and gastrointestinal protection are the main activities of *D. officinale* polysaccharide. The activity of *D. officinale* polysaccharides and the corresponding mechanism are shown in Figure 3.

### Immunomodulatory

**FIGURE 4:**
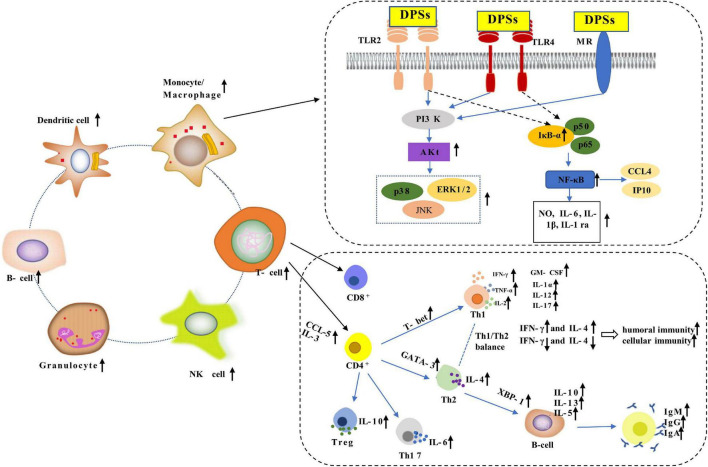
The potential immunomodulatory mechanisms of *D. officinale*.

Immune regulation is one of the most important physiological of *D. officinale* polysaccharides. It could regulate the immune system in animals by binding to receptors on the surface of immune cells and activating different signaling pathways. (44). The immunomodulatory mechanism of *D. officinale* polysaccharide was shown in Figure 4. *D. officinale* polysaccharide relieved immunosuppression by regulating the proportion and differentiation status of immune cells (such as CD4^+^ T cells, CD8^+^ T cells, B lymphocytes, macrophages, and natural killer cells) (1, 16, 28). In general, they could enhance the phagocytic ability of macrophages through non-specific immunomodulatory functions and promote the production of certain cytokines [such as interleukin-2 (IL-2), interleukin-6 (IL-6), interferon-γ (IFN-γ), tumor necrosis factor-α (TNF-α)] and other inflammatory factors [such as reactive oxygen species (ROS), nitric oxide (NO)] (17).

Huang et al. (60) studied the immunomodulatory activity of polysaccharides (DOP-1-1) isolated from the stems of *D. officinale*. The results suggested that DOP-1-1 induced an immune response through the nuclear factor-kappa B (NF-κB) mediated by TLR4 signaling pathway, confirming that CCL4 and IP10 may be new targets of DOP-1-1-stimulated immune response.

In one case, a polysaccharide (DOP-W3-b) with more intestinal immune activity was isolated from the stem of *D. officinale*. Oral administration showed that DOP-W3-b promoted Peyer’s patches (PPs) and mesenteric by changing the structure of the intestinal mucosa. Lymph nodes (MLNs) cytokines secreted IFN-γ and IL-4 effectively modulated intestinal mucosal immune activity and increased the production of secretory immunoglobulin A (sIgA) (39).

Two novel component polysaccharides (DLP-1 and DLP-2) were isolated from *D. officinale* leaves, both of which could stimulate the proliferation and phagocytosis of RAW 264.7 cells, and promote the secretion of NO, IL-6, IL-1β, and TNF-α. Moreover, the immunomodulatory activity of DLP-2 was higher than that of DLP-1 at the same concentration. Thus, DLP-1 and DLP-2 could be used as functional food additives (51). Similarly, DCP also has immunostimulatory activity *in vitro*, the mechanism of which was similar to that of DLP-1 and DLP-2 (53). Meanwhile, two component polysaccharides (DOP-1 and DOP-2) were isolated from *D. officinale* by DEAE cellulose and Sephacryl S-400, which caused a significant stimulation of cytokine secretion of both splenocytes and macrophages. DOP-1 had a greater effect on lymphocyte activation and DOP-2 on macrophage activation (64). In conclusion, the immunomodulatory effect of ferric polysaccharide was mainly achieved by inhibiting MAPK and NF-κB activation, promoting cytokine secretion and macrophages activity.

### Anti-tumor

Tumors are caused by different factors such as genetics, environment, and lifestyle. Tumorgenesis is a complex process, closely related to immune and inflammatory processes. Its anti-tumor activity mechanism is mainly to enhance immune regulation, inhibit tumor cell proliferation, and regulate tumor cell microenvironment (1, 65, 66).

**FIGURE 5:**
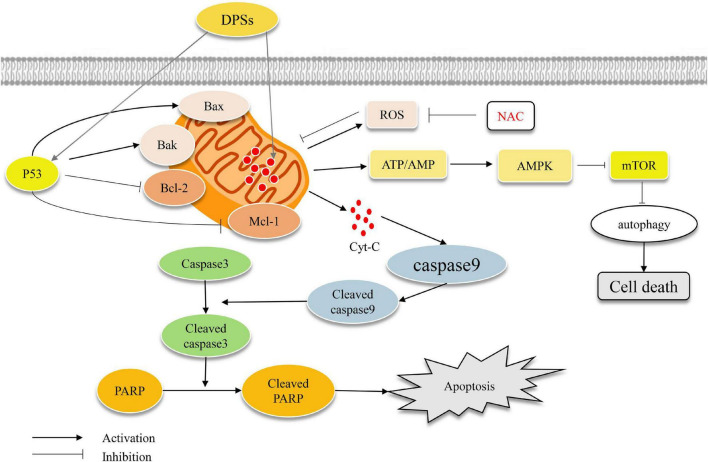
The potential anti-tumor mechanisms of *D. officinale*.

Wei et al. (49) determined the antitumor cell activity of purified DOPA-1 by cell experiments. They found that DOPA-1 inhibited the growth of HepG-2 cells in a dose-dependent manner. The levels of ROS in HepG-2 cells treated with DOPA-1 increased, the mitochondrial membrane potential decreased, and the expressions of P53, Bax, and Bak were up-regulated, and the expressions of Bcl-2 and Mcl-1 were down-regulated as shown in Figure 5. Liang et al. (9) found that DOPS could restore the intestinal barrier function and enhance the intestinal anti-tumor immune response, thereby inhibiting the occurrence of colorectal tumors. Xing et al. (35) reported that four component polysaccharides (DOP-40, DOP-50, DOP-60, and DOP-70) induced apoptosis in HepG-2 cells through Bcl-2 and Bax-dependent pathways. In addition, purified polysaccharides (DCPP-I, DCPP-I-a, and DCPP-II) showed stronger inhibitory effect on SPC-A-1 cell proliferation than crude polysaccharides (43).

Comparing the antitumor activities of different polysaccharides in DOP122, DOP20, DOP8, and DOP2, we found that DOP20 had the strongest inhibitory effect on the proliferation of osteosarcoma (OS) U2OS and Saos-2 cells (67). In addition, DOP20 promoted DDP-induced cell apoptosis by the mitochondrial pathway by up-regulates the expression of P53, Bax, Bak; down-regulated the expression of Bcl-2 and Mcl-1; increased the proportion of Cleaved caspase9/Caspase9, Cleaved caspase3/Caspase3, and Cleaved PARP/PARP (67). Besides, the anti-tumor activity of DOPs on OS cells was superior to that of mannose-containing monosaccharides and oligosaccharides, and the molecular weight was also an important factor affecting the anti-tumor activity. In the future, polysaccharides with relatively appropriate molecular weight or molecular weight range should be found for application.

### Hypoglycemic

**FIGURE 6:**
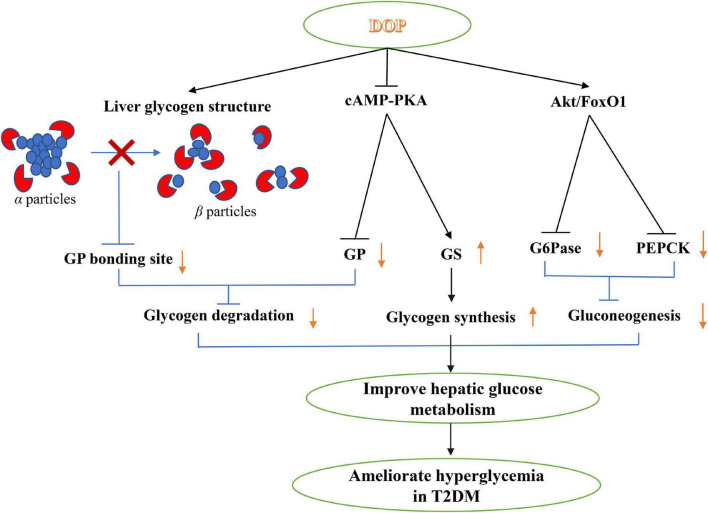
The potential hypoglycemic mechanisms of *D. officinale*.

Diabetes mellitus is a metabolic disease caused by insufficient secretion of the pancreas, characterized by elevated blood sugar levels (33). Studies have shown that *D. officinale* polysaccharides could promote insulin secretion and inhibit glucagon secretion, and achieve the purpose of extrapancreatic hypoglycemia by inhibiting epinephrine-induced hepatic glycogenolysis and promoting hepatic glycogen synthesis (17, 68, 69). Liu et al. (70) explored the hypoglycemic mechanism of DOP from the glucagon-mediated signaling pathway and the structure of hepatic glycogen that catalyzes hepatic glucose metabolism. The results showed that DOP significantly inhibited the glucagon-mediated cAMP-PKA signaling pathway, increased the GS expression and decrease GP expression, thereby promoting hepatic glycogen synthesis and inhibiting hepatic glycogen degradation. Furthermore, through the glucagon-mediated Akt/FoxO1 signaling pathway, the expression of G6Pase and PEPCK was reduced, and hepatic gluconeogenesis was inhibited in diabetic mice (Figure 6).

Taking alloxan-induced diabetic mice as the research object, the hypoglycemic activities of different varieties of *D. officinale* polysaccharides (DHP, DOP, DNP, and DCP) were compared. The results of serum FBG levels and Serum insulin level showed that DHP, DOP, and DNP had significant hypoglycemic activity (71). In addition, different doses of DOPs were fed to diabetic rats, and it was found that DOPs had obvious hypoglycemic activity and increased serum insulin and GLP-1 levels in diabetic rats. Moreover, The Ca^2+^/CaM/CaMKII and MAPK pathways were involved in DOP-induced GLP-1 secretion in STC-1 cells *in vitro* (50). Nowadays, long-term use of hypoglycemic drugs and insulin injections could cause serious side effects to the body. *D. officinale* polysaccharide had the effect of repairing islet cells, and the potential to become a new anti-diabetic drug in the future. However, a large number of clinical trials are still needed to study the hypoglycemic activity of *D. officinale* polysaccharide.

### Hepatoprotective

The liver is the largest digestive gland in the human body, a place for material metabolism, and an important detoxification organ to maintain the body’s homeostasis. Some drugs or chemical substances induce liver damage and cause various diseases including hepatitis, alcoholic liver injury, liver fibrosis, liver cancer, etc (72). In a male Sprague–Dawley rat model of alcoholic liver injury, after oral administration of *D. officinale* polysaccharide (DOP) 400 and 100 mg/kg, ASF content in serum and liver tissue decreased, inflammatory cytokines (such as IL-1β, IL- 6, and TNF-α) were significantly decreased, and NF-κB phosphorylation was inhibited. *In vitro*, DOP increased LO2 cell viability, inhibited LDH release, and decreased IL-1β, IL-6, and TNF-α secretion (73). Lin et al. (62) studied that DOP feeding for 30 days could protect liver injury induced by acetaminophen (APAP), and further studies showed that DOP exerted hepatoprotective effect by inhibiting oxidative stress and activating the Nrf2-Keap1 signaling pathway. A study by Wang et al. showed that *D. officinale* polysaccharide (DOP) could protect ccl4-induced liver fibrosis by maintaining intestinal homeostasis and inhibiting the activation of LPS-TLR4-NF-κB signaling pathway (74).

### Anti-oxidation

With the deepening of scientific research, it has been gradually recognized that many diseases are related to oxidative stress, such as diabetes, cardiovascular disease, hypertension, and other chronic diseases, so antioxidant strategies have received more and more attention. *D. officinale* contains high activity of superoxide dismutase (SOD), peroxidase (POD), catalase (CAT), and has good antioxidant activity *in vitro* and *in vivo* (3).

Huang et al. (75) compared the antioxidant activity of *D. officinale* polysaccharide crude and purified in cyclophosphamide-induced mice. The results showed that the bold and purified compounds had good antioxidant activities against CTX-induced oxidative stress, including increased liver and thymus indices, increased antioxidant enzyme activity, and decreased malondialdehyde (MND) levels in serum, thymus, and liver. Wang et al. (56) isolated two polysaccharides (DOP-1 and DOP-2) from fermented *D. officinale* polysaccharides, and their antioxidant activities were concentration-dependent in DPPH free radical and hydroxyl radical scavenging tests. And the hydroxyl radical scavenging rate of DOP-1 was better than that of DOP-2. Fan et al. (76) separated six polysaccharides from *D. officinale* by a step-by-step alcohol precipitation method. The antioxidant test *in vitro* showed that the six components all had strong antioxidant activities, and the smaller the molecular weight, the stronger the antioxidant activity.

### Gastrointestinal protection

Modern pharmacological studies indicated that plant polysaccharides protected gastrointestinal mucosa, regulate intestinal flora, and prevent colorectal cancer (1, 77). Studies have found that *D. officinale* polysaccharide has a protective effect on the gastrointestinal tract, and because it is not easily digested and absorbed, it has the effect of regulating intestinal flora (39, 78).

The polysaccharide fraction (LOP-1) isolated from *D. officinale* leaves had protective effect of ethanol-induced gastric mucosal injury (61). The results showed that the mechanism of action of LOP-1 was to activate the expression levels of p-AMPK, LC3β, and Beclin-1, inhibit the expression levels of p-mTOR and p62, and increase the proportion of Bcl-2 and Bax, thereby reduce gastric mucosal damage and pathological damage (61). Liang et al. (15) found that *D. officinale* polysaccharide (DOP-G-3) could reduce inflammatory damage, reduce colon pathological damage, and protect colon from dextran sulfate sodium (DSS)-induced colitis. Ma et al. (79) found that *D. officinale* polysaccharide could reduce the expression of IL-6, Raf-2, MEK1, MEK2, and ERK by promoting the expression of EGFR and TFF-1 expression, thus reducing the level of inflammatory factors and protecting the gastric mucosa.

Polysaccharides could not be directly digested, the behavior in the intestinal tract is considered a “ridge” between microbiota and host communication. The gastrointestinal tract is often referred to as the “second brain” of the human body. It was reported that the abundance of gut bacteria increased after 25 days of *D. officinale* (DOW-5b) treatment, especially beneficial microorganisms such as butyrate-producing Clostridium, Parabacteroides and anti-inflammatory *Akkermansia*, while harmful bacteria such as *Proteobacteria* decreased (80). In addition, when studying the effect of *D. officinale* (DOP) fermentation on human intestinal flora, the results showed that DOP could increase the abundance of *Firmicutes* and *Bacteroidetes*, and decrease the abundance of *Proteobacteria* (81). At the same time, the content of short-chain fatty acids (SCFA) mainly composed of acetic acid, propionic acid and butyric acid increased significantly (81). In conclusion, *D. officinale* polysaccharides could improve the disease and maintain physiological activities by increasing the diversity of human intestinal flora, regulating the proportion of intestinal flora, and promoting the growth and proliferation of beneficial flora.

### Other

Except as mentioned above biological activities, other biological activities of *D. officinale* polysaccharides were also evaluated. Yue et al. (55) isolated a novel sulfated polysaccharide (S32S) from *D. officinale* stems, which has obvious anti-angiogenic effects by inhibiting the migration and tube formation of human microvascular endothelial cells (HMEC-1). Moreover, *D. officinale* polysaccharide was also found to have neuroprotective activity. Feng et al. (82) proved that *D. officinale* polysaccharide (DOP) could alleviate the cognitive decline in SAMP8 mice by inhibiting the activation of microglia in the hippocampus of SAMP8 mice. Zhang et al. (83) found that fermented *D. officinale* polysaccharide (FDOP) had the effect of reducing antioxidant damage and anti-aging. The results showed that FDOP reduced antioxidant damage and anti-aging effects by upregulating Nrf2/Keap1 and (TGF-β)/Smads signaling pathways.

## Structure-function relationship

It is generally believed that the bioactivity of polysaccharides is closely related to their structural properties, including monosaccharide composition, molecular weight, chain conformation, and glycosidic bond types (44). Due to the complex structure of *D. officinale* polysaccharides and the limited understanding of the structure-activity relationship, it is of great significance to explore the structure-activity relationship of *D. officinale* polysaccharides for the development and application of *D. officinale* polysaccharides.

As we all know that the bioactivity of plant polysaccharides is mainly related to the monosaccharide composition, wherein the more complex the monosaccharide composition, the better the biological activity (3). Two polysaccharides rich in mannose (DOPA-1, DOPA-2) were isolated from *D. officinale*. Studies have shown that high mannose content has mild immunostimulatory activity and antioxidant activity (5). Liang et al. (84) isolated two novel polysaccharides (DOP1, DOP2) from *D. officinale* by the DES method, which consisted of glucose and mannose in molar ratios of 2.2:1 and 3.7:1, respectively. The study found that both components had strong antioxidant activity, and the higher the mannose content, the stronger the activity. Molecular weight was also a factor that affects the activity of *D. officinale* polysaccharides. Many scholars have found that the molecular weight of polysaccharides was positively correlated with activity, and polysaccharides with a molecular weight greater than 100 kDa show better activity (3, 16, 17). For example, Huang et al. (5) isolated two component polysaccharides (DOPA-1, DOPA-2) from *D. officinale* with molecular weights of 394 and 362 kDa, respectively, and the results showed that the higher molecular weight component DOPA-1 was more active. In addition, polysaccharide conformation and backbone may also affect polysaccharide biological activity (37, 44). Huang et al. (75) found that the structure containing (1→4)-β-D-Man*p* and O-acetyl was the main reason for the immune activity of polysaccharides.

In general, most studies on the structure-activity relationship of *D. officinale* polysaccharides are single rather than systematic. At present, scholars still need to conduct systematic research on the structure-activity relationship of *D. officinale* polysaccharides, which is conducive to the further development of polysaccharide preparations with specific functions.

## Conclusions and perspectives

With the study of biomacromolecules, such as nucleic acids and proteins, developing rapidly, our understanding of the crucial nature of polysaccharides has dramatically expanded over the last few decades. *D. officinale* polysaccharides have received extensive attentions due to their extremely high pharmacological effects and excellent biological properties. This paper summarizes the latest research progress of *D. officinale* polysaccharides, including the extraction, isolation and purification, structure analysis, biological activity, mechanism of action, and structure-activity relationship of *D. officinale* polysaccharides. Although there are many studies on *D. officinale* polysaccharides, there are still many problems to be solved in terms of the current research results.

First, the structurally active relationship of polysaccharides from *D. officinale* was not fully elucidated. Therefore, in-depth research on the structure analysis and structure-activity relationship of *D. officinale* polysaccharides is necessary; the accurate structures of polysaccharides are obtained by applying the latest science and technology to further study their biological activities; the structure-activity relationship database of *D. officinale* polysaccharides is constructed to eliminate the limitations of research. Secondly, the current structural analysis of *D. officinale* polysaccharides mainly focuses on the primary structure of monosaccharide composition, molecular weight, main chain and side chain, and further analysis of the advanced structure is required. Third, with the study of new technologies “omics,” such as metabolomics, microbiomics, proteomics, genomics, transcriptomics, and bioinformatics are widely used in the study of biological activity mechanisms. Fourth, the immune activity of polysaccharides and gut microbiota has received extensive attention. The current mainstream opinion is that the relative molecular mass and polymer length affect the participation of polysaccharides in the recognition and glycolysis processes between microorganisms. It is necessary to strengthen the study of the relationship between the structure of natural polysaccharides and their regulation of intestinal microecology. The human race is experiencing a novel coronavirus outbreak and investigators are eager to find an immunopotentiator to deal with the virus problem.

In summary, *D. officinale* polysaccharides have great application prospects in the fields of biology, medicine, functional food and so on.

## Author contributions

YH, HC, BC, HG, and YY contributed to the conception and design of the study. YH wrote the first draft of the manuscript. GC, WH, and ZJ wrote the sections of the manuscript. LL and YY contributed to the funding of the study and writing – review and editing. All authors contributed to the article and approved the submitted version.

## Conflict of Interest

The authors declare that the research was conducted in the absence of any commercial or financial relationships that could be construed as a potential conflict of interest.

## Publisher’s Note

All claims expressed in this article are solely those of the authors and do not necessarily represent those of their affiliated organizations, or those of the publisher, the editors and the reviewers. Any product that may be evaluated in this article, or claim that may be made by its manufacturer, is not guaranteed or endorsed by the publisher.
